# Role of cytochrome *c* in α-synuclein radical formation: implications of α-synuclein in neuronal death in Maneb- and paraquat-induced model of Parkinson’s disease

**DOI:** 10.1186/s13024-016-0135-y

**Published:** 2016-11-24

**Authors:** Ashutosh Kumar, Douglas Ganini, Ronald P. Mason

**Affiliations:** Free Radical Biology Group, Immunity, Inflammation, and Disease Laboratory, National Institute of Environmental Health Sciences, National Institutes of Health, 111 T.W. Alexander Dr., Research Triangle Park, Durham, NC 27709 USA

**Keywords:** Cytochrome *c*, α-synuclein radical, Peroxidase, Parkinson’s disease, Immuno-spin trapping

## Abstract

**Background:**

The pathological features of Parkinson’s disease (PD) include an abnormal accumulation of α-synuclein in the surviving dopaminergic neurons. Though PD is multifactorial, several epidemiological reports show an increased incidence of PD with co-exposure to pesticides such as Maneb and paraquat (MP). In pesticide-related PD, mitochondrial dysfunction and α-synuclein oligomers have been strongly implicated, but the link between the two has not yet been understood. Similarly, the biological effects of α-synuclein or its radical chemistry in PD is largely unknown. Mitochondrial dysfunction during PD pathogenesis leads to release of cytochrome *c* in the cytosol. Once in the cytosol, cytochrome *c* has one of two fates: It either binds to apaf1 and initiates apoptosis or can act as a peroxidase. We hypothesized that as a peroxidase, cytochrome *c* leaked out from mitochondria can form radicals on α-synuclein and initiate its oligomerization.

**Method:**

Samples from controls, and MP co-exposed wild-type and α-synuclein knockout mice were studied using immuno-spin trapping, confocal microscopy, immunohistochemistry, and microarray experiments.

**Results:**

Experiments with MP co-exposed mice showed cytochrome *c* release in cytosol and its co-localization with α-synuclein. Subsequently, we used immuno-spin trapping method to detect the formation of α-synuclein radical in samples from an in vitro reaction mixture consisting of cytochrome *c*, α-synuclein, and hydrogen peroxide. These experiments indicated that cytochrome *c* plays a role in α-synuclein radical formation and oligomerization. Experiments with MP co-exposed α-synuclein knockout mice, in which cytochrome *c*-α synuclein co-localization and interaction cannot occur, mice showed diminished protein radical formation and neuronal death, compared to wild-type MP co-exposed mice. Microarray data from MP co-exposed wild-type and α-synuclein knockout mice further showed that the absence of α-synuclein per se or its co-localization with cytochrome *c* confers protection from MP co-exposure, as several important pathways were unaffected in α-synuclein knockout mice.

**Conclusions:**

Altogether, these results show that peroxidase activity of cytochrome *c* contributes to α-synuclein radical formation and oligomerization, and that α-synuclein, through its co-localization with cytochrome *c* or on its own, affects several biological pathways which contribute to increased neuronal death in an MP-induced model of PD.

**Electronic supplementary material:**

The online version of this article (doi:10.1186/s13024-016-0135-y) contains supplementary material, which is available to authorized users.

## Background

Parkinson’s disease (PD) is a prominent progressive neurological disorder with more than ten million cases all across the globe [1]. PD involves the loss of dopaminergic neurons in the substantia nigra pars compacta [2]. This degeneration is associated with motor disturbances including tremor, rigidity, and bradikinesia [3, 4]. Although the cause of sporadic PD is not fully understood, various factors, including heavy metals and pesticides, have been implicated [5, 6]. Epidemiological studies also show that the risk of PD in humans is strongly increased by combined exposure to the fungicide Maneb (manganese ethylene-1,2-bisdithiocarbamate) and the herbicide paraquat (1,1-dimethyl-4,4-bipyridinium) [7, 8]. These two pesticides are commonly used concurrently in agriculture. Maneb and paraquat together (MP) are well documented as a trigger that induces the PD phenotype in mice [3, 9]. Therefore, we used the Maneb- and paraquat-induced PD phenotype as a model system for our experiments.

The pathological features of PD include an abnormal accumulation of the α-synuclein throughout various brain regions in the remaining dopaminergic neurons [10]. Certain mutations in SNCA (the gene which encodes α-synuclein) or post-translational oxidative modifications have been speculated to increase the propensity of α-synuclein to oligomerize and accumulate. Although α-synuclein oligomers [11, 12] play crucial roles in neuronal regulation during PD pathogenesis, the underlying initiating mechanism of oligomerization remains unknown [13, 14]. We propose that α-synuclein radical formation might be a key mechanism that initiates its oligomerization. In one of our recent publications we showed the formation of α-synuclein radicals in the mid-brain of MP co-exposed mice [15]. Since MP co-exposure is known to trigger oxidative stress by complex 1 and/or complex 3 inhibition [16], mitochondrial dysfunction [16], and NADPH oxidase activation [17, 18], a range of possibilities existed for how α-synuclein radical was formed. Furthermore, in pesticide-related PD, mitochondrial dysfunction and α-synuclein oligomers have been strongly implicated [19, 20], but the underlying mechanism of α-synuclein oligomerization and the link between the two has not yet been understood. Similarly, with the detection of α-synuclein oligomers from the brains of PD patients, the involvement of α-synuclein in PD pathogenesis is well established [21–23]; however, its role in the disease process and its effects on critical cellular pathways which contribute to neuronal death remain unknown. There is considerable evidence that free radicals, mitochondrial dysfunction, and α-synuclein oligomerization play roles in the pathogenesis of PD [21, 22, 24–26]. Within dopaminergic neurons, mitochondria are in peril of damage from reactive oxygen species generated by disruption of the electron transport chain or autoxidation of dopamine [20, 27]. Damage to presynaptic mitochondria, caused by environmental insults such as pesticides, results in the release of cytochrome *c*. Once in the cytosol, cytochrome *c* has one of two fates: It either binds to apaf1 and initiates the apoptotic cascade or can act as a peroxidase [28]. This peroxidase activity of cytochrome *c* is triggered either through cleavage of methionine-80 from heme iron by action of proteolytic enzymes present in cytosol or through its oxidation [29–32]. Theoretically, cytochrome *c* as a peroxidase can form radicals on proteins in proximity using hydrogen peroxide as a substrate [29, 33, 34]. As one of the most abundant proteins in the terminals of dopaminergic neurons, α-synuclein in the presence of anionic phospholipids can easily divert cytochrome *c* and, hence, prevent binding of cytochrome *c* to apaf1 [35–38]. In addition, α-synuclein has a very high affinity for anionic phospholipids such as phosphatidylserine, and these lipids can facilitate its crosslinking to cytochrome *c* [28, 39].

Owing to reports showing co-localization of cytochrome *c* and α-synuclein in the brains of PD patients [28], we hypothesized the role of cytochrome *c* as a peroxidase in α-synuclein radical formation. Altogether, we aimed to investigate (I) a possible correlation between mitochondrial dysfunction concurrent with cytochrome *c* release and the role of cytochrome *c* in α-synuclein radical formation, (II) the role of cytochrome *c* in α-synuclein oligomerization, and (III) the biological implications of α-synuclein radical formation or the role of α-synuclein per se in terms of its effects on biological pathways and neuronal death.

Here we report for the first time that cytochrome *c* plays a crucial role in α-synuclein radical formation and oligomerization and that α-synuclein radical formation or α-synuclein per se significantly affects several biological pathways, which ultimately contributes to neuronal death in the MP-induced mouse model of PD. This understanding will be fundamental to determine the role of peroxidase chemistry in PD pathogenesis. Understanding cytochrome *c*-mediated α-synuclein radical formation and its aftereffects in a pesticide-induced model of PD will provide new insights into the role of protein radicals and α-synuclein in PD pathogenesis.

## Methods

### Chemicals

Cytochrome *c*, Maneb (Manganese ethylene-1,2-bisdithiocarbamate), paraquat (1,1-dimethyl-4,4-bipyridinium), paraformaldehyde, recombinant α-synuclein, Triton X-100, and TRI Reagent were from Sigma (St. Louis, MO). 5, 5-dimethyl-1-pyrroline *N*-oxide (DMPO) was obtained from Dojindo Laboratories (Rockville, MD) and used without further purification. Chicken polyclonal and mouse monoclonal anti-DMPO antibodies were developed in our laboratory and used in the immuno-spin trapping studies. Rabbit polyclonal anti-tyrosine hydroxylase antibody was from Millipore (Billerica, MA). Rabbit anti-α-synuclein, mouse anti-cytochrome *c*, anti-complex IV, anti-caspase-3, anti-caspase-9 and anti-β-actin antibodies were from Abcam (Cambridge, MA). Micro BCA Protein Assay Kit, Permount, RIPA buffer, Surfact-Amps X-100, Mitochondria Isolation Kits for Tissue, and Pierce Classic Immunoprecipitation Kits were from Thermo Scientific (Rockford, IL). Nitrocellulose membranes, Prolong Gold anti-fade reagent with DAPI and AlexaFluor secondary antibodies were from Invitrogen (Grand Island, NY). The Vectastain Elite ABC kit for immunohistochemistry was from Vector Laboratories (Peterborough, UK). RNeasy mini kit columns were from Qiagen (Valencia, CA). Agilent Whole Mouse 4X44K microarray slides and the reagents used in microarray experiments were all from Agilent Technologies (Santa Clara, CA).

### Animal treatment

Adult (8-10-week-old), pathogen-free male mice (C57BL6/J from Jackson Laboratories, Bar Harbor, ME) were housed one to a cage for a week for acclimatization before experimental dosing. Mice that contained disrupted α-synuclein (α-synuclein knockout mice, stock number 003692) were treated similarly. Age-matched, wild-type C57BL6/J mice that contained α-synuclein served as the control animals for knockout experiments. The animals were housed under standard conditions of temperature and humidity with a 12 h light/dark cycle and fed a standard pellet diet and water *ad libitum*. Animals were treated with normal saline (control), paraquat (10 mg/kg, ip) and Maneb (30 mg/kg, ip) twice a week for 6 weeks. A minimum of 3–5 animals were used per group for Western blot, immunohistochemical, and confocal studies. All animals were treated in strict accordance with the NIH Guide for the Care and Use of Laboratory Animals, and the animal study proposal was approved by the NIEHS ASP review board.

### In vivo immuno-spin trapping studies

DMPO was administered at a dose of 2 g/kg, ip, two hours after the final dose of MP at the 6-week time point in a set of animals in which protein radical detection experiments were performed. Animals were sacrificed 2 h after DMPO injection, and sections/samples were used for confocal microscopy and immunoprecipitation experiments to detect α-synuclein radicals.

### Confocal microscopy of brain tissue

Cryocut sections (30 μm thick) were collected and standard staining protocols were used as published earlier [15]. In brief, tissue slices were permeabilised (0.2% Surfact-Amps X-100 in PBS) for 5 min and blocked overnight and then incubated for 2 h with the primary anti-DMPO (1:2,000) and/or anti-α-synuclein (1:2,000) and/or anti-cytochrome c (1:2000) and anti-tyrosine hydroxylase antisera (1:4,000), followed by incubation with secondary Alexa Fluor antisera diluted 1:1,000 for an hour. Slides were washed, dried and mounted using Prolong Gold anti-fade reagent with DAPI. Confocal images were taken on a Zeiss LSM 710-UV meta microscope (Carl Zeiss Inc. Oberkochen, Germany) using a Plan-NeoFluar 40X/1.3 Oil DIC objective.

### Immunoprecipitation and Western blot analysis

Immunoprecipitation of α-synuclein adducted to DMPO from nigrostriatal tissue homogenates was carried out with the Pierce Classic Immunoprecipitation Kit using a standard protocol as described elsewhere [15]. For Western blot, standard protocols were followed with the use of mouse monoclonal anti-DMPO antibody (1:250)/ anti- α-synuclein antibody (1:3000)/anti-tyrosine hydroxylase antibody (1:4,000)/ anti-cytochrome c (1:4000)/anti-β -actin antibodies (1:4,000), and bands were detected using the appropriate fluorescent secondary antibody (1:10,000, diluted in washing buffer) labeled with infrared dyes. An Odyssey infrared imaging system (Li-Cor Biosciences, Lincoln, NE) was used to acquire the images. For Western blotting of cytochrome *c*, we isolated mitochondrial and cytosolic fractions using a Thermo Scientific Mitochondria Isolation Kit for Tissue with the standard Dounce homogenization and differential centrifugation method. The resulting mitochondrial and cytosolic fractions were analyzed by Western blot for cytochrome *c* and complex IV. A Western blot for complex IV, which is a mitochondrial marker, was used to rule out any possible traces of mitochondria in the cytosolic fraction.

### Chemical reactions

Typically, a reaction mixture of 1.5 μM α-synuclein, 0.5 μM cytochrome *c*, 50 mM DMPO and hydrogen peroxide in 100 mM Chelex-treated sodium phosphate buffer (pH 7.4) with 25 μM DTPA in a total volume of 200 μl was incubated for 1 h at 37 °C. Samples were diluted and used for electrophoresis and immuno-spin trapping experiments using an anti-DMPO antibody (Fig. 3a). This reaction mixture was also analyzed using cytochrome *c* and α-synuclein antibody to test the oligomerization of α-synuclein (Fig. 3b).

**Fig. 1:**
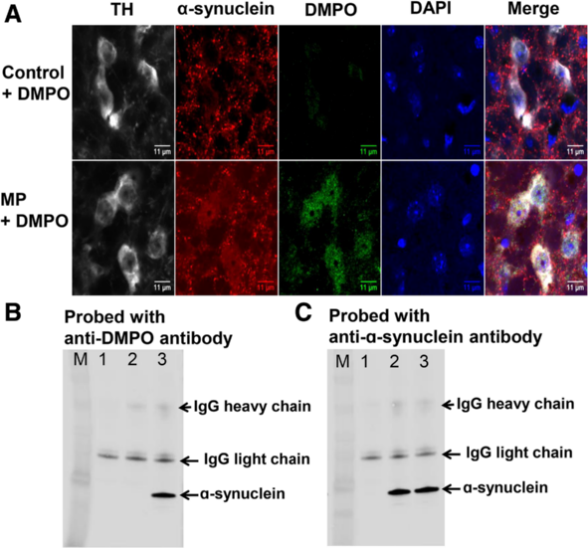
Detection of α-synuclein-centered radicals in tyrosine hydroxylase positive neurons. **a** Confocal images of mid-brain sections from sham + DMPO (control) and MP + DMPO-treated mice showing the co-localization of anti-DMPO, α-synuclein and tyrosine hydroxylase. **b** Immunoprecipitation of DMPO adducts from nigrostriatal tissue homogenates of control (lane 1 and 2) and MP co-exposed (lane 3) mice. Proteins were immunoprecipitated from nigrostriatal tissue homogenates using either normal mouse IgG (lane1) or with anti-α-synuclein antibody (lane 2 and 3) followed by Western blotting of immunoprecipitates using rabbit polyclonal anti-DMPO antibody. **c** Proteins were immunoprecipitated from nigrostriatal tissue homogenates as in figure B, followed by Western blotting of immunoprecipitates using rabbit polyclonal anti-alpha-synuclein antibody. Blots are representative of four different immunoblots from an equal number of experiments

**Fig. 2:**
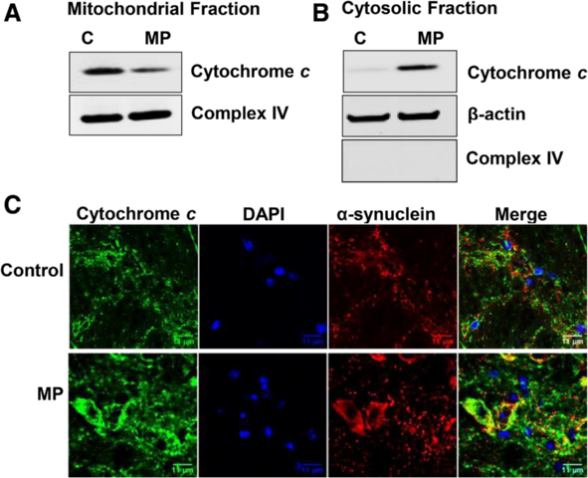
Western blot of cytochrome *c* with respective constitutive proteins from (**a**) mitochondrial and (**b**) cytosolic fraction of nigrostriatal tissue of control and MP co-exposed mice. **c** Confocal images showing the co-localization of cytochrome *c* and α**-**synuclein in mid-brain slices of control and MP co-exposed mice. Blots or images are representative of four different immunoblots from an equal number of experiments

**Fig. 3:**
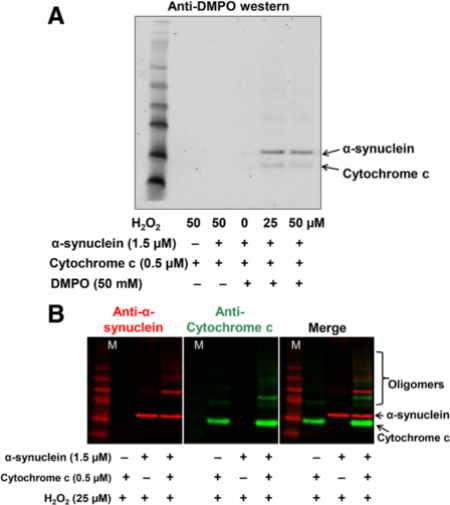
**a** Anti-DMPO Western blotting showing α-synuclein radical formation. The reaction mixture containing α-synuclein (1.5 μM), cytochrome c (0.5 μM), and DMPO (50 mM) in the presence and absence of various concentrations of hydrogen peroxide (0, 25, 50 μM) was incubated at 37 °C for 1 h in chelexed phosphate buffer with 25 μM DTPA in a total volume of 200 μl. Samples were then used for electrophoresis and immuno-spin-trapping experiments using an anti-DMPO antibody. **b** Western blotting showing oligomerization of α-synuclein using the same reaction mixture and conditions as in A

### Immunohistochemistry

For immunohistochemistry of tyrosine hydroxylase (TH), mice were anesthetized and perfused transcardially with 4% paraformaldehyde. Perfused mouse brains were excised, post-fixed overnight at 4 °C, and cryoprotected in sucrose (10–30%). Serial coronal sections (30 μm thick) were collected to ensure capture of the entire SN/VTA region. Sections were then immunostained using a standard protocol [40]. The sections were mounted using Permount, and images were captured with a bright field microscope at 8× magnification. Coded slides were used, and counting was performed independently to ensure unbiased counting of TH-positive neurons in every 3rd serial section. TH-positive neurons were counted and analyzed bilaterally using a Metamorph image analysis tool [15]. A minimum of five animals per group were used for counting TH-immunoreactive neurons.

### Global gene expression by microarray and bioinformatics data analyses

Total RNA was isolated from the nigrostriatal tissue of three independent triplicates using the TRIzol (Ambion, Life Technologies, Grand Island, NY) and RNeasy mini kit columns with in-column DNAse treatment (Qiagen, Valencia, CA). Gene expression analysis was conducted using Agilent whole mouse Genome 4x44 multiplex arrays (Product # G4846A, Agilent Technologies, CA). Total purified RNA samples were labelled with Cy3 according to the manufacturer’s protocol. Initial data were obtained using the Agilent Feature Extraction software (v. 12) and further processed using R (version 3.1.3) and RStudio (version 0.98.1103). Raw microarray data was normalized, and the background was subtracted using the limma package (version 3.22.6). Principal component analyses were performed. Gene annotation was performed using the database mgug4122a.db. Differential expression was evaluated. Weighted Venn diagrams were drawn using the Venn diagram package (version 1.6.0). Heatmaps were prepared using the gplot package (version 2.16.0). Two different 2-contrast GSEA analyses were conducted: 1. MP-treated versus control; 2. MP-treated α-synuclein knockout versus control mice. GSEA analyses were performed using the full table of annotated genes (log transformed intensities) with the hallmark database, h.all.v5.0.symbols, 1,000 permutations, and defaults for other parameters. Only gene sets with normalized *p*-value (NORM p-val) lower than 0.05 and false discovery rate (FDR q-val) lower than 0.25 were considered enriched.

### Statistical analyses

All animal experiments were repeated at least three times with a minimum of 3–5 mice per group (*n* = 3–5). One-way analysis of variance (ANOVA) was used for statistical analysis. The Newman–Keuls Post-Test was used for multiple comparisons. The results are expressed as mean ± SEM. The differences were considered statistically significant at *p* < 0.05.

## Results

### Maneb and Paraquat co-exposure induces α-synuclein radical formation in dopaminergic neurons of exposed mice

Immuno-spin trapping experiments (Additional file 1: Scheme S1) in conjunction with confocal microscopy with the midbrain sections of MP co-exposed mice showed anti-DMPO staining and its co-localization with α-synuclein and tyrosine hydroxylase, which is a marker enzyme for dopaminergic neurons (Fig. 1a). This result indicated that protein radicals were formed within dopaminergic neurons and perhaps on α-synuclein. To test for α-synuclein radical formation, α-synuclein was immunoprecipitated from nigrostriatal tissue homogenates of controls and MP co-exposed mice and probed with anti-DMPO antibody (Fig. 1b) and anti- α-synuclein antibody (Fig. 1c). Proteins immunoprecipitated with normal mouse IgG did not show either detectable DMPO adducts (lane 1, Fig. 1b) or α-synuclein (lane 1, Fig. 1c). α-Synuclein was detected in immunoprecipitates from both control and MP co-exposed groups (lanes 2 and 3, Fig. 1c); however, an anti-DMPO-positive band on the immunoprecipitated α-synuclein (lane 3, Fig. 1b) was present only in the MP co-exposed group. This further indicates that MP co-exposure led to α-synuclein radical formation in dopaminergic neurons. Having confirmed that MP co-exposure led to α-synuclein radical formation, we further investigated its possible correlations with peroxidase function of cytochrome *c*, which typically comes out in cytoplasm after some extent of mitochondrial damage [9, 41].

### Cytochrome c and α-synuclein co-localization

Mitochondrial damage/dysfunction is often associated with cytochrome *c* release in cytoplasm; therefore, cytochrome *c* levels were analyzed by Western blotting, which showed increased levels of cytochrome *c* in the cytosolic fraction of nigrostriatal tissue of MP co-exposed mice (Fig. 2a). Subsequent confocal microscopy experiments showed co-localization of cytochrome *c* with α-synuclein in the mid-brain of MP co-exposed mice (Fig. 2b). These results were consistent with cytochrome *c* leaking out into the cytosol and co-localizing with α-synuclein. Cytochrome *c* acting as a peroxidase may form radicals on proteins in its proximity. Based on the cytochrome *c*-α-synuclein co-localization, the known peroxidase activity of cytochrome *c* in the cytoplasm, and the presence of hydrogen peroxide as a peroxidase substrate in dopaminergic neurons of MP co-exposed mice, we decided to further investigate the possible role of cytochrome *c* in α-synuclein radical formation using a pure in vitro system.

### Cytochrome c as a peroxidase induces α-synuclein radical formation and oligomerization

To investigate the role of cytochrome *c* in α-synuclein radical formation, an in vitro reaction mixture was used. The reaction mixture contained α-synuclein (1.5 μM), cytochrome *c* (0.5 μM), and DMPO (50 mM) in the presence and absence of hydrogen peroxide (0, 25–50 μM), and was incubated at 37 °C for 1 h in chelexed phosphate buffer with DTPA (25 μM). Samples were then used for immuno-spin trapping experiments using an anti-DMPO antibody and results indicated protein radical formation on α-synuclein and to a much lesser extent on cytochrome *c* (Fig. 3a). The dependence of radical formation on hydrogen peroxide indicated the role of cytochrome *c* as a peroxidase in α-synuclein formation. Subsequent experiments showed that conditions similar to those that led to protein radical formation on α-synuclein also led to oligomerization of α-synuclein (Fig. 3b). Interestingly, oligomerization of α-synuclein was not seen when cytochrome *c* was absent in the reaction mixture. Together, these results showed the role of cytochrome *c* as a peroxidase in α-synuclein radical formation and oligomerization. Owing to the higher availability of the peroxidase substrate hydrogen peroxide in dopaminergic neurons of MP co-exposed mice, these results indicate the important role of cytochrome *c* as a peroxidase in PD pathogenesis.

### α-Synuclein plays a key role in protein radical formation in dopaminergic neurons

**Fig. 4:**
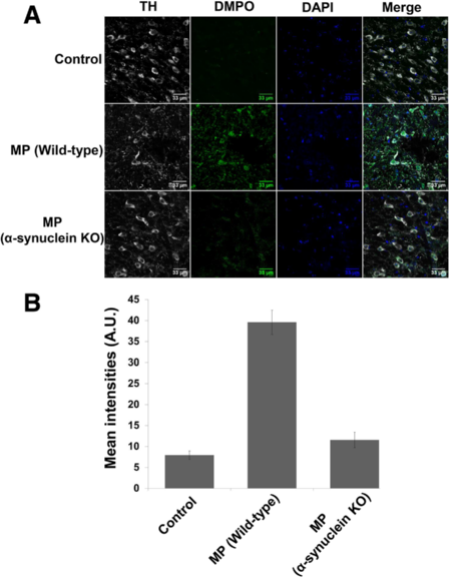
Protein radical formation in the midbrain of mice after 6 weeks of Maneb (30 mg/kg, i.p.) and paraquat (10 mg/kg, i.p.) co-exposure. **a** Confocal images showing anti-DMPO staining in mid-brain slices of Maneb and paraquat co-exposed mice. **b** Fluorescence intensity quantification of anti-DMPO staining

After confirming protein radical formation on α-synuclein (α-synuclein radical) and elucidating the underlying mechanism of its formation, we further analyzed the role of α-synuclein in overall protein radical formation in the dopaminergic neurons of MP co-exposed mice. In order to ascertain the role of α-synuclein, α-synuclein knockout mice were co-exposed to MP for 6 weeks and then analyzed using immuno-spin trapping [42]. Immuno-spin trapping experiments in conjunction with confocal microscopy of mid-brain sections of MP co-exposed mice showed intense anti-DMPO staining, which indicates protein radical formation. Furthermore, experiments with MP co-exposed α-synuclein knockout mice showed diminished protein radical formation compared to wild-type MP co-exposed mice (Fig. 4a, b). These results indicated the role of α-synuclein in overall protein radical formation. In α-synuclein knockout mice, cytochrome *c*-α-synuclein co-localization cannot occur, and in that case cytochrome *c* may support apoptosis instead of acting as a peroxidase and contributing to protein radical formation. This was one possible explanation of the diminished protein radical formation in MP co-exposed α-synuclein knockout mice; however, to get a clear picture of what is different in α-synuclein knockout mice, we further investigated its effects on biological pathways using microarrays.

### Microarray analysis shows diminished biological effects of MP in α-synuclein knockout mice

**Fig. 5:**
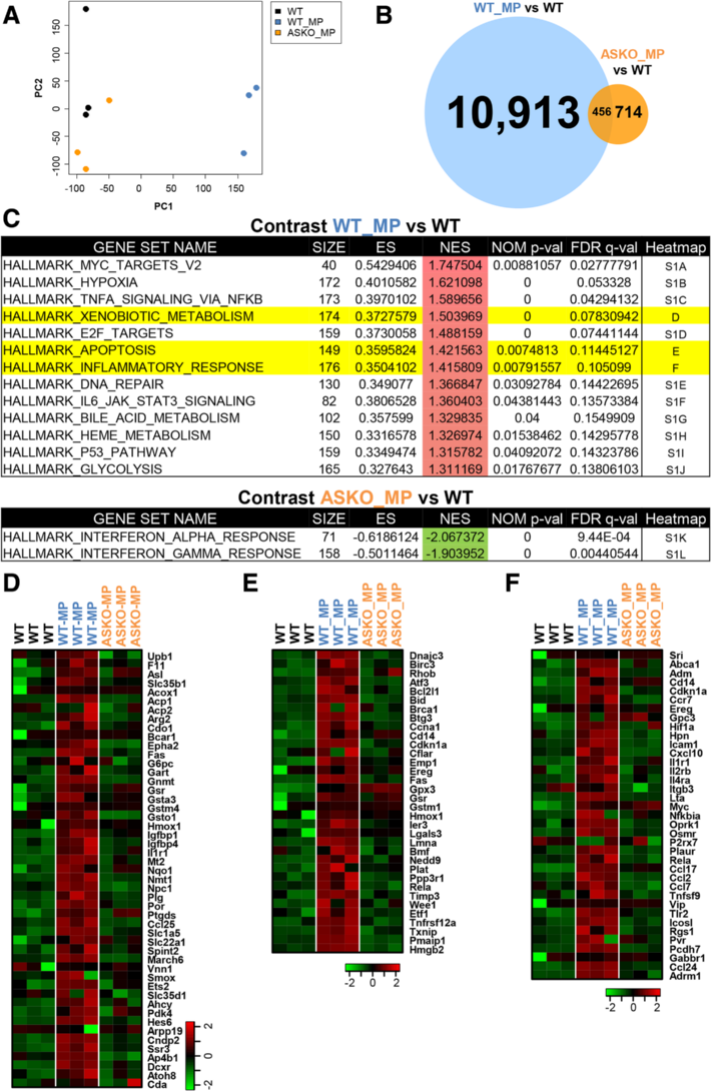
Microarray analysis of nigrostriatal tissue of MP co-exposed wild type and α-synuclein knockout mice. **a** Graph showing nearly similar gene expression pattern of wild-type sham-treated mice and α-synuclein knockout mice co-exposed to MP for 6 weeks. **b** Weighted Venn diagram for total number of genes with significantly different expression values (*p*-value lower than 0.05) in nigrostriatal tissue of treated animals compared to wild-type controls. **c** Upper panel showing upregulation of 13 pathways in wild type MP co-exposed mice compared to wild type sham-treated mice; lower panel showing downregulation of 2 pathways in MP co-exposed α-synuclein knockout mice compared to wild-type sham-treated mice. Heat maps for all these pathways are shown in Additional file 1: Figure S1. **d** Heat map showing changes in expression of hallmark genes in the xenobiotic metabolism pathway. **e** Heat map showing changes in expression of hallmark genes in the apoptotic pathway. **f** Heat map showing changes in expression of hallmark genes in the inflammatory response pathway. ES stands for Enrichment Score, and NES stands for Normalized Enrichment Score

Analyses of variance for global gene expression of nigrostriatal tissue showed that MP co-exposure to wild-type animals, which have α-synuclein, induced a massive change in global gene expression (Fig. 5a); in contrast, in nigrostriatal tissue of α-synuclein knockout animals MP treatment induced only minor changes in global gene expression. Genes differentially expressed (*p*-values lower than 0.05) in wild-type animals co-exposed with MP were more than 94% of all genes differentially expressed, while less than 10% of all differentially expressed genes were altered in the α-synuclein knockout animals (Fig. 5b). GSEA analyses of microarray data showed major changes in 13 gene sets under the same conditions as those that produced α-synuclein radical formation in wild-type MP co-exposed mice (Fig. 5c). These gene sets were related to the hallmark pathways of MYC targets, hypoxia, TNF α signaling, xenobiotic metabolism, E2F targets, apoptosis, inflammation, DNA repair, JAK-STAT3 signaling, bile acid metabolism, heme metabolism, the P53 pathway, and glycolysis (Additional file 1: Figure S1A-S1J). We show heatmaps for the expression levels of several genes that play important roles in xenobiotic metabolism, inflammation and apoptosis (Fig. 5d–f). Simultaneously, MP co-exposed α-synuclein knockout mice showed decrease in genes of only two pathways (Interferon α and Interferon Gamma) after the same MP co-exposure regimen as in wild type mice. Since these pathways were downregulated, it was apparent that these changes were also protective in nature (Fig. 5c). Altogether, these results indicate that the absence of α-synuclein per se or α-synuclein radical formation diminished the biological effects on several pathways that contribute to increased free radical generation and neuronal death.

### Maneb and paraquat co-exposure-induced neuronal death is diminished in α-synuclein knockout mice

**Fig. 6:**
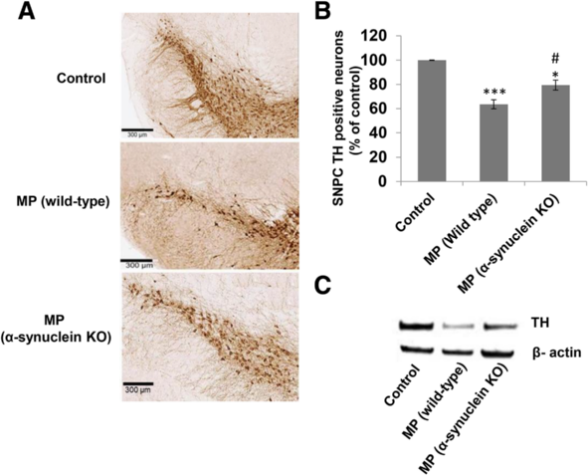
Tyrosine hydroxylase (TH) immunoreactivity in the substantia nigra of mouse brains after 6 weeks of MP co-exposure. **a** TH immunoreactivity in frozen brain sections of controls, MP co-exposed wild type, and MP co-exposed α-synuclein knockout mice. **b** Number of TH positive neurons in the substantia nigra pars compacta (SNPC) region of all experimental groups as in **a**. **c** Western blot analysis of TH protein in the nigrostriatal tissue of mouse brains following 6 weeks of Maneb and paraquat co-exposure. *p-value < 0.05, ***p-value < 0.001 as compared with control and # *p*-value < 0.05 as compared with the MP co-exposed group

To further investigate the role of radical chemistry and the effects of α-synuclein radical formation on neuronal death, tyrosine hydroxylase immunoreactivity was investigated. After 6 weeks of exposure, we observed a significant decline in tyrosine hydroxylase immuno-reactivity and the number of TH-positive neurons in the substantia nigra pars compacta in the brains of wild-type mice. The absence of α-synuclein conferred neuroprotection with diminished neuronal death induced by MP co-exposure in α-synuclein knockout mice (Fig. 6a–c). This concurrence between neuronal death and protein radical formation (Fig. 4a, b) and its reliance on α-synuclein, as evident with diminished neuronal death and protein radical formation in MP co-exposed α-synuclein knockout mice, indicates an important role of α-synuclein in the neuronal death process.

## Discussion

Based on our previous published work [15] and results presented here, we report that there are two distinct mechanisms which can lead to α-synuclein radical formation. One is α-synuclein radical formation through NADPH oxidase activation and peroxynitrite formation, and the other is through peroxidase activity of cytochrome *c*. Since MP co-exposure leads to both NADPH oxidase activation and mitochondrial dysfunction, both pathways can lead to α-synuclein radical formation. However, the fact remains that both pathways are distinct because radical formation through NADPH oxidase is mediated by peroxynitrite, mostly diffusing out from microglia [15], whereas, cytochrome *c* mediated α-synuclein radical formation starts from leaky mitochondria within dopaminergic neurons.

As one of the most abundant proteins in the terminals of dopaminergic neurons, α-synuclein in the presence of anionic phospholipids can easily divert cytochrome *c* and, hence, prevent binding of cytochrome *c* to apaf1 [35]. α-Synuclein has a very high affinity for anionic phospholipids such as phosphatidylserine, and these lipids can facilitate its cross linking to cytochrome *c* [28, 39]. The cross-linking of α-synuclein to cytochrome *c* in the cytosol delays initiation of apoptosis in neurons [28]. Because cytochrome *c*-dependent formation of apoptosomes and activation of caspases designates a point of no return in the apoptotic program, the interaction of cytochrome *c* with α-synuclein after its release from mitochondria confers immediate protection. However, this vital function of diverting cytochrome *c* from initiating apoptosis is accompanied by the emergence of peroxidase activity of the cytochrome *c*. Thus, protection against acute apoptotic cell death comes with a penalty: the formation of a complex (cytochrome *c* and α-synuclein) with persistent peroxidase activity which can further affect proteins such as α-synuclein as well as others. Theoretically, peroxidase-induced protein radical formation should not be limited only to α-synuclein, but rather to a large extent depends on the protein’s concentration, affinity or proximity to cytochrome *c*, presence of oxidizable amino acid residues, and the availability of peroxidase substrates [28, 29, 39, 43].

For cytochrome *c* to work as a peroxidase, it must leak out into the cytoplasm, where it gets activated by oxidation or cleavage of its methionine residue by action of the proteolytic enzymes present in the cytosol [28, 31]. This cytochrome *c* that can work as a peroxidase in cytoplasm was co-localized with α-synuclein in mid-brain slices of MP co-exposed mice (Fig. 2). To further investigate the mechanism and confirm the role of cytochrome *c* in α-synuclein radical formation, we used an analogous in vitro system that co-incubated cytochrome *c*, α-synuclein and hydrogen peroxide. Based on the in vitro results, we inferred that cytochrome *c* as a peroxidase can form radicals on α-synuclein (Fig. 3a). Owing to the presence of oxidizable residues [21, 44] (tyrosine, cysteine and histidine) [45] on α-synuclein, it is highly likely that similar chemistry takes place in the dopaminergic neurons of MP co-exposed mice, due in part to the higher abundance of peroxidase substrates in dopaminergic neurons.

Several groups have reported that, physiologically, α-synuclein exists almost exclusively in its unfolded monomeric form, and oligomeric forms are mostly associated with diseases [19, 46]. Biochemical measurements of α-synuclein isolated from brains of PD patients revealed the presence of covalently linked α-synuclein oligomers [47], but what triggers this covalent oligomerization had not yet been understood. Therefore, we further investigated the oligomerization of α-synuclein in the presence and absence of cytochrome *c* and hydrogen peroxide and found that α-synuclein radical formation and oligomerization was taking place concurrently (Fig. 3b). This can be explained in the light of the fact that a protein as a free radical can serve as an initiating mechanism for dimerization or can simply form dimers with another protein radical. Reports in the literature show that α-synuclein has four tyrosine residues [48], and formation of tyrosyl radicals is well known to lead to radical-radical recombination to form dityrosine and, thereby, dimer formation [21]. These reports support our present data that cytochrome *c* in the presence of hydrogen peroxide is capable of forming dimers of α-synuclein through radical-radical dimerization. Also radical formation on α-synuclein can alter the orientation of hydrophobic moieties of α-synuclein, and that can make the protein sticky and promote its aggregation [45]. Furthermore, radical formation on cytochrome *c*, though to a lesser extent (Fig. 3a), and its oligomerization (Fig. 3b), is in accordance with reports in which authors have shown oxidative modifications and oligomerization of cytochrome c by hydrogen peroxide [49]. In addition, cytochrome *c* has also been documented to show properties of self-association and oligomerization [50], which might be another reason for cytochrome *c* oligomerization in our experimental set up (Fig. 3b).

After confirming the role of cytochrome *c* in α-synuclein radical formation and oligomerization, we further investigated the role of α-synuclein in overall protein radical formation using α-synuclein knockout mice. A significant decline in overall protein radical formation as evidenced by the diminished intensity of anti-DMPO in α-synuclein knockout mice was due to the fact that in knockout mice, cytochrome *c*-α-synuclein co-localization can’t occur. The absence of co-localization actually diverts cytochrome *c* into the apoptotic pathway, and then it no longer contributes to protein radical formation. This is one possible explanation of diminished protein radical formation in MP co-exposed α-synuclein knockout mice; however, microarray data indicated that α-synuclein crucially affects several pathways which may contribute to diminished protein radical formation in MP co-exposed knock out mice.

**Scheme 1:**
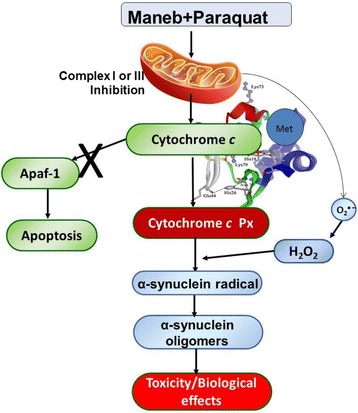
Role of cytochrome *c* as peroxidase in α-synuclein radical formation and oligomerization

Microarray analysis showed that in wild-type MP co-exposed mice where α-synuclein was present, 13 important biological pathways were upregulated. However, in α-synuclein knockout mice, MP co-exposure could elicit downregulation of only two pathways (Fig. 5c). These results support the importance of α-synuclein in animal models of PD, and PD pathogenesis in general. Although thirteen (Additional file 1: Figure S1A–S1K) pathways showed significant changes, we focused our attention on xenobiotic metabolism, inflammatory response and apoptotic pathways (Fig. 5d–f). These pathways were upregulated only in animals with α-synuclein. Since these pathways rely on or contribute to free radical generation [22, 45], we can further corroborate the role of α-synuclein in overall protein radical generation and PD pathogenesis. Furthermore, Western blots for caspase-9 and cleaved caspase-3 (Additional file 1: Figure S2) indicated that the extent of apoptosis is certainly high in MP co-exposed mice. However, it was close to control levels in MP co-exposed α-synuclein knockout mice. These results are consistent with microarray data in Fig. 5e, in which it is shown that while the overall gene expression pattern of “hallmark of apoptosis genes” is similar between the controls and MP co-exposed α-synuclein knockout mice, it is significantly higher in MP co-exposed wild-type mice. These results appeared to be in line with previous reports, which showed that α-synuclein protects neurons from apoptosis downstream of free-radical production through modulation of the MAPK signaling pathway [51]. As shown by Bayir et al., [28] and in this manuscript, α-synuclein diverts cytochrome *c* from the initial acute apoptosis. However, this immediate protection comes with the formation and accumulation of α-synuclein oligomers (Scheme 1). As shown by several investigators, these oligomers can further impair several important rescue processes like autophagy [52, 53] and make cells more prone to accumulate damage. This eventually leads to delayed apoptosis, and that is why we see upregulation of markers of apoptosis in wild type mice after chronic MP co-exposure for 6 weeks. Typically, Parkinson’s disease is characterized by slow and progressive neuronal death, therefore the cytochrome *c*- and α-synuclein-mediated process of accumulating damage and conferring delayed apoptosis makes better sense in the disease context. This explanation is further supported by the fact that the absence of α-synuclein in knockout mice confers protection against chronic MP co-exposure (Fig. 6a–c) [54, 55]. These results appear to be in line with another study in which mice lacking α-synuclein showed decreased loss of striatal dopamine following prolonged chronic MPTP administration [23].

## Conclusion

In summary, this study provides evidence for the first time that cytochrome *c* as a peroxidase plays a role in α-synuclein radical formation and oligomerization. Also, through various lines of evidence, this study demonstrates the involvement of α-synuclein in overall protein radical formation, alterations in several biological pathways and dopaminergic neuronal death. Altogether, the detection of α-synuclein radicals and understanding the underlying involvement of cytochrome *c* as a peroxidase in PD pathogenesis will expand our current knowledge about the mechanistic aspects of PD.

## Additional file

Additional file 1: This contains Figure S1, S2, Scheme S1. Figure S1 contains heat maps showing changes in gene expression as mentioned in Fig. 5c. Figure S2 shows Western blots for α-synuclein, caspase-9, and cleaved caspase-3. Scheme S1 shows an outline of immuno-spin trapping method in which anti-DMPO antibody is used to detect protein radicals. (PDF 361 kb)

## Abbreviations

DMPO: 5,5-dimethyl-1-pyrroline *N*-oxide
MP: Maneb and paraquat
TH: Tyrosine hydroxylase
