# Supplementary material for: Role of cytochrome c in α-synuclein radical formation: implications of α-synuclein in neuronal death in Maneb- and paraquat-induced model of Parkinson’s disease
Source: Mol Neurodegener. 2016 Nov 24;11:70. doi: 10.1186/s13024-016-0135-y (PMC5122029; doi:10.1186/s13024-016-0135-y)
Supplement: Additional file 1: — This contains Figure S1, S2, Scheme S1. Figure S1 contains heat maps showing changes in gene expression as mentioned in Fig. 5c. Figure S2 shows Western blots for α-synuclein, caspase-9, and cleaved caspase-3. Scheme S1 shows an outline of immuno-spin trapping method in which anti-DMPO antibody is used to detect protein radicals. (PDF 361 kb) [file 13024_2016_135_MOESM1_ESM.pdf]

Supplementary Figure S1 (A-L)

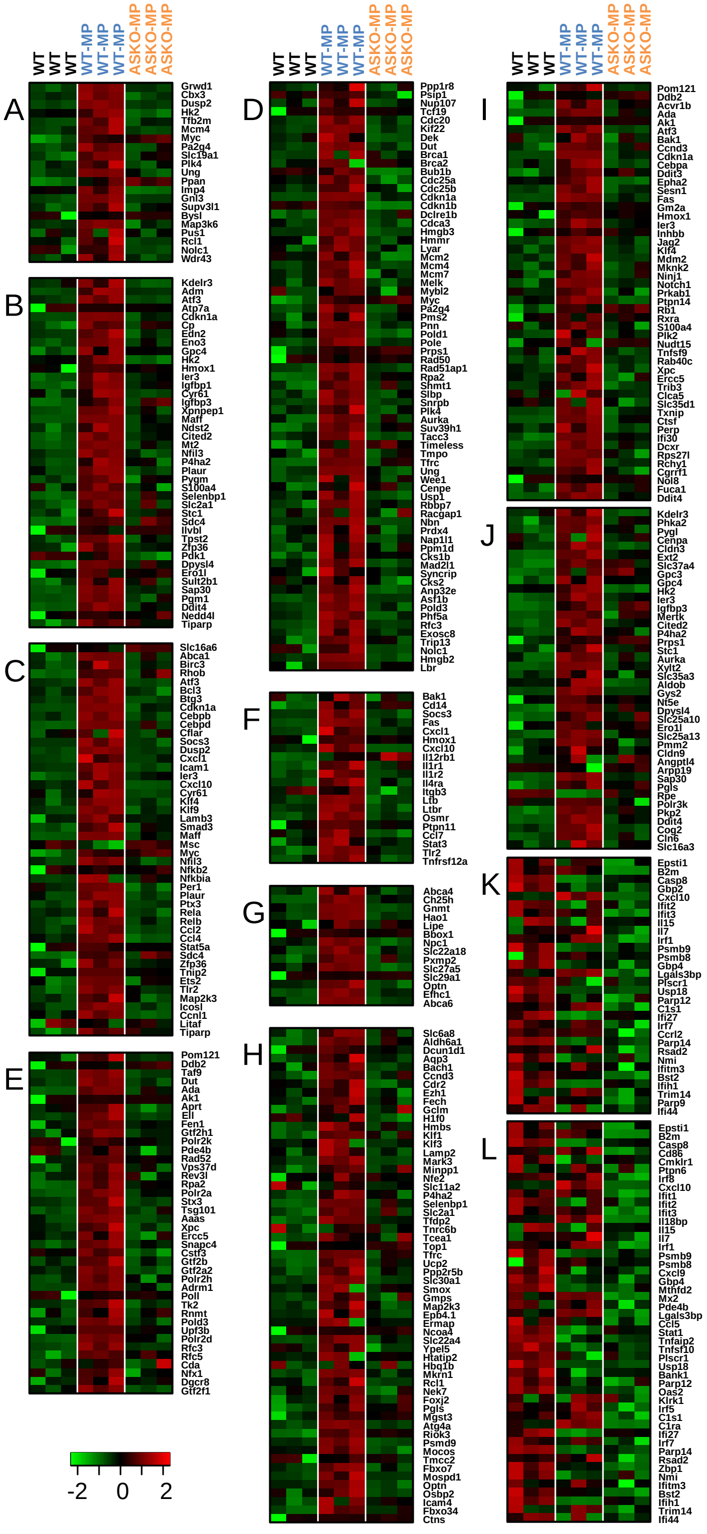

Supplementary Figure S1 (A-L) - Heat maps showing changes in gene expression in hallmark pathways of MYC targets (S1A), hypoxia (S1B), TNF α signaling (S1C), E2F targets (S1D), DNA repair (S1E), JAK-STAT3 signaling (S1F), bile acid metabolism (S1G), heme metabolism (S1H), the P53 pathway (S1I), glycolysis (S1J), Interferon α (S1K), and interferon gamma (S1L).

Supplementary Figure S2

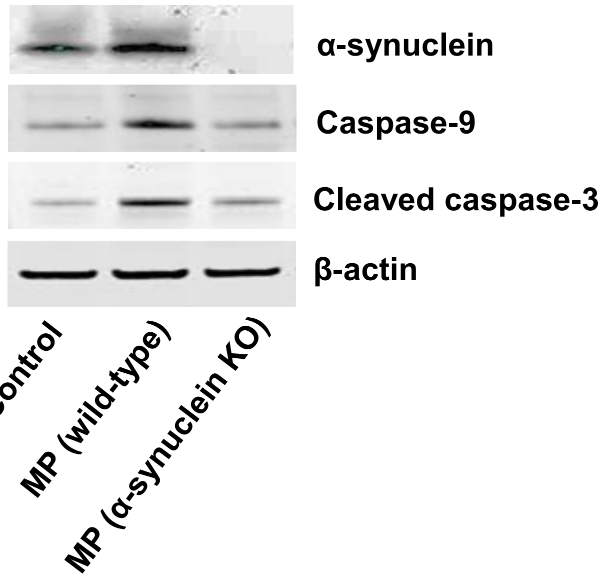

Supplementary Figure S2- Western blots showing changes in protein levels of α-synuclein, caspase-9, and cleaved caspase-3 in nigrostriatal tissue homogenates from controls, and MP co-exposed wild-type and α-synuclein knockout mice.

Supplementary Scheme 1

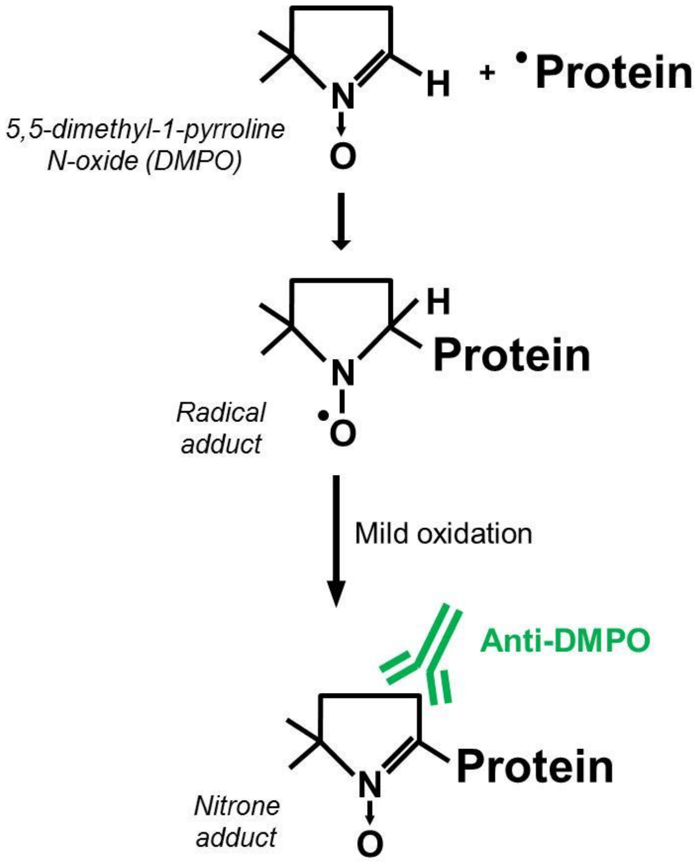

Supplementary Scheme 1- Scheme showing an outline of immuno-spin trapping method in which anti-DMPO antibody is used to detect protein radicals.
